# Analysis of the Seasonal Fluctuation of γδ T Cells and Its Potential Relation with Vitamin D_3_

**DOI:** 10.3390/cells11091460

**Published:** 2022-04-26

**Authors:** Birthe Bernicke, Nils Engelbogen, Katharina Klein, Jeanette Franzenburg, Christoph Borzikowsky, Christian Peters, Ottmar Janssen, Ralf Junker, Ruben Serrano, Dieter Kabelitz

**Affiliations:** 1 Institute of Immunology, University Hospital Schleswig-Holstein (UKSH) Campus Kiel, 24105 Kiel, Germany; birthebernicke@web.de (B.B.); katharina.klein@uksh.de (K.K.); christian.peters@uksh.de (C.P.); ottmar.janssen@uksh.de (O.J.); 2 Institute of Clinical Chemistry, University Hospital Schleswig-Holstein (UKSH) Campus Kiel, 24105 Kiel, Germany; nils.engelbogen@uksh.de (N.E.); jeanette.franzenburg@uksh.de (J.F.); ralf.junker@uksh.de (R.J.); 3 Institute of Bioinformatics and Statistics, University Hospital Schleswig-Holstein (UKSH) Campus Kiel, 24105 Kiel, Germany; c.borzikowsky@gmx.de

**Keywords:** calcitriol, cytokine production, cytotoxicity, flow cytometry, gamma/delta T cells, immunophenotyping, seasonal fluctuation, vitamin D_3_

## Abstract

In addition to its role in bone metabolism, vitamin D_3_ exerts immunomodulatory effects and has been proposed to contribute to seasonal variation of immune cells. This might be linked to higher vitamin D_3_ levels in summer than in winter due to differential sun exposure. γδ T cells comprise a numerically small subset of T cells in the blood, which contribute to anti-infective and antitumor immunity. We studied the seasonal fluctuation of γδ T cells, the possible influence of vitamin D_3_, and the effect of the active metabolite 1α,25(OH)_2_D_3_ on the in vitro activation of human γδ T cells. In a retrospective analysis with 2625 samples of random blood donors, we observed higher proportions of γδ T cells in winter when compared with summer. In a prospective study over one year with a small cohort of healthy adults who did or did not take oral vitamin D_3_ supplementation, higher proportions of γδ T cells were present in donors without oral vitamin D_3_ uptake, particularly in spring. However, γδ T cell frequency in blood did not directly correlate with serum levels of 25(OH)D_3_. The active metabolite 1α,25(OH)_2_D_3_ inhibited the in vitro activation of γδ T cells at the level of proliferation, cytotoxicity, and interferon-γ production. Our study reveals novel insights into the seasonal fluctuation of γδ T cells and the immunomodulatory effects of vitamin D_3_.

## 1. Introduction

γδ T cells account for approximately 5% of CD3 T cells in human peripheral blood. In contrast to the major populations of CD4 and CD8 T cells expressing the conventional αβ T cell receptor (TCR), the germ line TCR repertoire of γδ T cells is very small. There are only six expressed Vγ genes and a similarly small number of Vδ genes. Among the γδ T cells in peripheral blood, most express Vγ9 paired with Vδ2, while other subsets (e.g., Vδ1) are usually rare in blood but more abundant in mucosal tissues [1]. However, the proportion of γδ T cells and their subset distribution varies greatly in the peripheral blood of healthy adult donors and is influenced by age and gender [2,3,4]. Vγ9Vδ2 T cells (referred to as Vδ2 in the following) recognize pyrophosphate molecules (“phosphoantigens” (pAg)) independently of HLA class I or class II molecules. However, recognition of such pAg, which are secreted by many microbes but can also be produced by tumor cells, is absolutely dependent on members of the butyrophilin (BTN) family of transmembrane molecules, notably BTN3A1 and BTN2A1 [5,6,7]. The production of endogenous pAg, such as isopentenyl pyrophosphate (IPP), can be massively stimulated by nitrogen-containing aminobisphosphonates (e.g., Zoledronate (ZOL)), which block an enzyme in the mevalonate pathway leading to upstream accumulation of IPP [8,9]. In view of their HLA-independent tumor cell recognition and their potent cytotoxic activity, γδ T cells have recently attracted great interest as potential effector cells in cell-based cancer immunotherapy [10,11,12]. In addition, however, γδ T cells can also exert regulatory functions [13,14], and the potential involvement of γδ T cells in autoimmune diseases has been discussed [15,16].

Vitamin D_3_ is an essential regulator of calcium and phosphate metabolism and thus of bone homeostasis. In addition, immunoregulatory properties of vitamin D_3_ have been identified, and various diseases spanning from autoimmunity and chronic inflammation to some infections have been associated with vitamin D_3_ deficiency [17,18]. Vitamin D_3_ can affect both innate and adaptive immunity and appears to inhibit Th1 and favor Th2 T cell responses [19,20,21,22,23]. Low vitamin D_3_ levels favor inflammatory conditions and Th17 T cell differentiation associated with an increased incidence of autoimmune diseases [19,24]. During exposure to sunlight, 7-dehydrocholesterol in the skin absorbs UV-B radiation and is converted to previtamin D_3,_ which then isomerizes to vitamin D_3_. This is sequentially metabolized in the liver to 25(OH)D_3_ and in the kidney to the biologically active metabolite 1α,25(OH)_2_D_3_ (1,25(OH)_2_D_3_ in the following) by 25-hydroxyvitamin D-1-α-hydroxylase (CYP27B1) [25,26]. Insufficient endogenous production of biologically active 1,25(OH)_2_D_3_ resulting from poor sunlight exposure (e.g., during the winter season) can be compensated by oral supplementation with vitamin D_3_ (cholecalciferol). The recommendations for adequate serum levels of 25(OH)D_3_ vary to some extent, but levels of <20 µg/L are considered inappropriately low and require oral supplementation [18].

Previous studies have shown that seasonal variations in serum levels of vitamin D_3_ are associated with a fluctuation in the subset distribution of peripheral blood T cells [27]. Moreover, it has previously been reported that the active vitamin D_3_ metabolite 1,25(OH)_2_D_3_ modulates the in vitro activation of human γδ T cells [28]. However, a systematic analysis of the seasonal variation of circulating γδ T cells and the possible correlation with vitamin D_3_ has not yet been performed. To address this issue, our study was designed to comprise three parts: (i) a retrospective analysis of the seasonal proportion of Vδ2 γδ T cells among CD3 T cells in a large cohort of random blood donors; (ii) a prospective study of γδ T cells and other immune cell subsets over a one-year period in a small group of healthy donors who did or did not take oral vitamin D_3_; and (iii) an in vitro analysis of the effects of 1,25(OH)_2_D_3_ on γδ T cell activation at the level of proliferation, cytotoxic activity, and cytokine production. 

## 2. Materials and Methods

**Blood samples**. Leukocyte concentrates obtained from healthy adult blood donors were provided by the Institute of Transfusion Medicine, University Hospital Schleswig-Holstein (UKSH) Campus Kiel. From the years 2011 to 2020, all samples obtained on a weekly basis were regularly screened for the proportion of total γδ T cells and Vδ2 γδ T cells among CD3^+^ T lymphocytes. These data were used in the retrospective analysis to calculate the proportion of γδ T cells in a large cohort of random healthy blood donors without consideration of age and sex. Such leukocyte concentrates were also used to isolate peripheral blood mononuclear cells (PBMC) for the analysis of the effects of 1,25(OH)_2_D_3_ on the activation and effector function of γδ T cells. In a prospective study, EDTA blood and serum were collected from 31 healthy adult blood donors every four to eight weeks for one year. The study participants were grouped according to their oral vitamin D_3_ uptake pattern into three groups: (i) no vitamin D_3_ (14 donors), (ii) interrupted vitamin D_3_ (no uptake mainly during late spring to early autumn (summer); 8 donors), and (iii) regular vitamin D_3_ (uptake throughout the year; 9 donors). Oral vitamin D_3_ (cholecalciferol) dosage ranged from a calculated average of 500 to 20,000 (mean 1877 ± 1968) IU per day. The actual schedule varied among donors. Information on the gender and age distribution of the study group is presented in Supplementary Table S1. The studies were performed in accordance with the declaration of Helsinki. The use of leukocyte concentrates from random donors for in vitro analysis and the study protocol of the prospective study have been approved by the Ethics Committee of the Medical Faculty of Christian-Albrechts University Kiel (code 405/10 and D579/19).

**Determination of 25(OH)D_3_ serum levels.** Serum levels of 25(OH)D_3_ (calcifediol) were measured using UHPLC as part of the routine diagnostic at the Institute of Clinical Chemistry, UKSH. Blood samples were centrifuged for 10 min at 3000× *g* to collect the serum. Prior to UHPLC analysis, the serum samples were prepared using kit reagents and sample preparation procedures from RECIPE (Munich, Germany). During the first step, 200 µL of the serum sample was treated with 200 µL of precipitation reagent to reduce the matrix load of the sample. Afterward, 200 µL of the organic solution containing the internal standard was added to extract vitamin D from the sample. After mixing and centrifugation, 5 µL of the upper phase, which contains the concentrated vitamin D, was injected into the UHPLC system. For the UHPLC analysis, a Chromaster HPLC-system (VWR, Radnor, PA, USA) with an isocratic pump, autosampler, column heater, and a UV detector was used. Then, 25(OH)D_3_ was chromatographically separated under isocratic conditions with a flow rate of 0.7 mL/min using the column and mobile phase provided with the reagent kit. The column temperature was kept at 35 °C, and the backpressure stayed below 300 bar. The injection interval between samples was 3 min with a retention time of 25(OH)D_3_ of 1.6 min. Peak detection is performed at a UV wavelength of 264 nm. Quantification of 25(OH)D_3_ was achieved by comparing its peak area to the peak area of the internal standard (retention time 2.1 min), which was added during sample preparation and behaved similarly to the analyte. The normal range of serum levels of 25(OH)D_3_ was defined as >20 µg/L [29].

**Flow cytometry.** Immunophenotyping in the prospective study was performed with EDTA blood as part of the routine diagnostic protocol in the Institute of Clinical Chemistry, UKSH Campus Kiel, and included relative proportions and absolute cell counts of CD3, CD4, CD8, CD19, and CD14 cells. Further subset analysis of γδ T cells was performed on Ficoll-Hypaque density gradient-separated peripheral blood mononuclear cells (PBMC). The following mAb were obtained from BioLegend (San Diego, CA, USA): anti-CD3-BV605 (clone OKT3), anti-CD3-PE (clone SK7), and anti-TCR Vδ2-FITC (clone B6). Anti-CD3-PE/APC (clone SK7), anti-TCR γδ-PECy7 (clone 11F2), anti-IFN-γ-PE (clone 4S.B3), and IgG1-PE were from BD Biosciences (Heidelberg, Germany). Anti-Vδ2-FITC (clone IMMU389) was obtained from Beckman Coulter (Krefeld, Germany). Anti-TCR γδ-FITC (clone 11F2) and anti-TCR Vδ2-VioBlue (clone REA771) were from Miltenyi Biotech (Bergisch Gladbach, Germany), and Anti-TCR Vγ9-FITC (clone 7A5) was generated in our laboratory [30]. For cell surface staining, 4 × 10^5^ cells were washed, stained in V-bottom microtiter plates for 20 min on ice with mAb, washed twice, and resuspended in 1% paraformaldehyde. FcR blocking reagent (Miltenyi Biotech) was added at 1:20 dilution before staining. For intracellular staining, cells were washed with staining buffer and stained with antibodies for cell surface CD3 and Vγ9. Subsequently, cells were permeabilized using Cytofix/Cytoperm kit (BD Biosciences) before staining with fluorochrome-conjugated anti-IFN-γ mAb or isotype control. All analyses were measured on a FACS-Canto or LSR-Fortessa cytometer (BD Biosciences), using DIVA (Data-Interpolating Variational Analysis) for acquisition and FlowJo™ v10.6.1 (Ashland, OR, USA) for data analysis. 

**Cell culture and measurement of γδ T cell proliferation**. To generate short-term expanded γδ T cell lines, PBMC were stimulated at 1 × 10^6^ cells/mL in 6-well plates with 2.5 μM zoledronate (ZOL) and 50 IU/mL IL-2 in the absence or presence of 1,25(OH)_2_D_3_ (Sigma Aldrich, Taufkirchen, Germany, or Enzo Life Sciences, Lörrach, Germany). Zoledronate and recombinant human IL-2 (Proleukin) were kindly provided by Novartis (Basel, Switzerland. A 50 µM stock solution of 1,25(OH)_2_D_3_ was prepared in DMSO and stored at −20 °C. The DMSO solvent control (1:1000) corresponding to the highest concentration of 1,25(OH)_2_D_3_ did not have any effect. IL-2 was added every other day, and cell cultures were split when required. The purity of γδ T cell lines after 14 d was routinely 74 to 92%. Cell cultures were subjected to microscopic inspection, and photographs were taken at ×100 magnification with an Axiovert 10 microscope (Leitz, Wetzlar, Germany) equipped with an Axiocam 105 camera device and ZEN 2 core v2.5 software (Zeiss, Oberkochen, Germany). For intracellular analysis of cytokine expression, 4 × 10^5^ freshly isolated PBMC were cultured for 48 h in 96-well round-bottom microtiter plates in the presence or absence of 2.5 µM ZOL and 50 nM 1,25(OH)_2_D_3_, and 3 μM monensin (Sigma Aldrich) was added during the last 4 h to prevent cytokine secretion. The culture medium was RPMI 1640 (Thermo Fisher Scientific) supplemented with antibiotics (100 U/mL penicillin, 100 µg/mL streptomycin) and 10% of heat-inactivated fetal bovine serum. All cell cultures were incubated at 37 °C in a humidified atmosphere of 5% CO_2_. Absolute numbers of viable γδ T cells per microculture well were measured after 8 d by a flow cytometry-based method termed standard cell dilution assay (SCDA), as described previously [31]. Briefly, cultured cells from 96-well round-bottom plates were washed and stained with anti-Vγ9-FITC mAb. After one washing step, cells were resuspended in a sample buffer containing a defined number of fixed standard cells and 0.2 μg/mL propidium iodide (PI). Standard cells were purified CD4 T cells that had been stained with APC-labeled antibodies and thereafter had been fixed in 1% paraformaldehyde. Based on the known number of standard cells (FITC^−^PI^+^APC^+^), the absolute number of viable Vγ9 T cells (FITC^+^PI^−^) in a given microculture well was determined as described previously [31,32]. The expansion rate was calculated in relation to the absolute number of Vγ9 T cells measured in ZOL- or HMBPP-stimulated cultures in the absence of 1,25(OH)_2_D_3._

**Measurement of interferon-γ in cell culture supernatants.** Short-term γδ T cell lines expanded for 14 d were washed and plated at 2 × 10^5^ cells/well in 96-well round-bottom plates coated or not with 0.5 µg anti-CD3 mAb OKT3 (BioLegend) per well or stimulated with 10 nM (*E*)-4-Hydroxy-3-methyl-but-2-enyl pyrophosphate (HMBPP; Echelon Biosciences, Salt Lake City, UT, USA). Then, 50 nM 1,25(OH)_2_D_3_ was added as indicated. After 24 h, cell-free supernatants were collected and stored at −20 °C until use. IFN-γ was quantified by ELISA with the DuoSet ELISA Kit from R&D Systems (Wiesbaden, Germany), following the manufacturer’s instructions. In each experimental setting, two replicates were included.

**Cytotoxicity measured by Real-Time Cell Analyzer**. Cytotoxic effector activity of short-term expanded γδ T cell lines against U251MG glioblastoma and BxPC3 pancreatic ductal adenocarcinoma target cells was determined by xCELLigence Real-Time Cell Analyzer (RTCA; Agilent, Santa Clara, CA, USA) which measures the decrease in the impedance of adherent tumor cells over extended time periods as a correlate of cell lysis. U251MG (ECACC 89081403) was obtained from the European Collection of Authenticated Cell Cultures (ECACC, Salisbury, UK). BxPC3 [33] was provided by Dr. Christian Röder, Institute for Experimental Cancer Research (UKSH Kiel, Germany). Tumor cell lines were maintained in a complete culture medium, and 0.05% trypsin/0.02% EDTA or accutase (Thermo Fisher Scientific) was used to detach adherent cell lines from flasks. RTCA was performed as previously described [34,35]. Briefly, 8000 tumor cells in complete medium were added to each well of the micro-E plates. After overnight incubation, γδ effector T cells were added at an effector/target ratio of 10:1 with or without HMBPP as a positive control to enhance TCR-dependent lysis. The impedance of the cells was recorded via electronic sensors on the bottom of the 96-well micro-E-plate every 3 min for up to 48 h. Results were analyzed with RTCA software (version 2.0.0.1301; Agilent, Santa Clara, CA, USA) and normalized, as described in [34]. Results of several experiments with different γδ T cell lines are summarized as the percentage of cell death induced by γδ T cells at various time points in relation to the corresponding tumor cell index in medium and Triton-X100 (maximal lysis). Time point zero was defined as the first measurement after the addition of γδ T cells.

**Statistical analysis.** All analyses were performed with the Graphpad Prism 8 (GraphPad Software, San Diego, CA, USA) and SPSS 28.0 software (IBM, Armonk, NY, USA). Linear regression was performed for the seasonal analysis of γδ T cells in the retrospective analysis. Statistical comparisons between groups were made using the Wilcoxon matched pairs signed-rank test for dependent samples without a normal distribution (RTCA, ELISA, SCDA). The Mann–Whitney *U* test was used in the case of the non-normal distribution of independent data in two groups. Non-normal data sets with more than two groups were analyzed with Kruskal–Wallis test and Dunn’s multiple comparison test. Levels of significance were set as * *p* < 0.05, ** *p* < 0.01, *** *p* < 0.001, and **** *p* < 0.0001.

## 3. Results

Our study consisted of three parts: (i) a retrospective analysis over ten years of the proportion of γδ T cells in a large cohort of random healthy blood donors; (ii) a prospective immunophenotypic analysis of γδ T cells and other immune cells in a small cohort of healthy adults who did or did not take oral vitamin D_3_ supplementation; and (ii) in vitro studies on the modulation of γδ T cell activation by the active vitamin D metabolite 1,25(OH)_2_D_3_. 

### 3.1. Retrospective Analysis

As a first approach to investigate a possible seasonal fluctuation in the proportion of γδ T cells circulating in peripheral blood, we performed a retrospective analysis of data from the weekly screening of leukocyte concentrates from random healthy adult blood donors. Parameters such as age, gender, or potential vitamin D_3_ uptake were not taken into consideration. From 2011 to 2020, a total of 2625 blood samples were stained for CD3/pan-γδ and CD3/Vδ2, and the proportion of total γδ T cells and Vδ2 T cells among CD3 T lymphocytes was calculated. The gating strategy used in this analysis is shown in Supplementary Figure S1. The results with the mean values per month of each year are displayed for total γδ T cells in Supplementary Figure S2a and for Vδ2 T cells in Supplementary Figure S2b.

**Figure 1:**
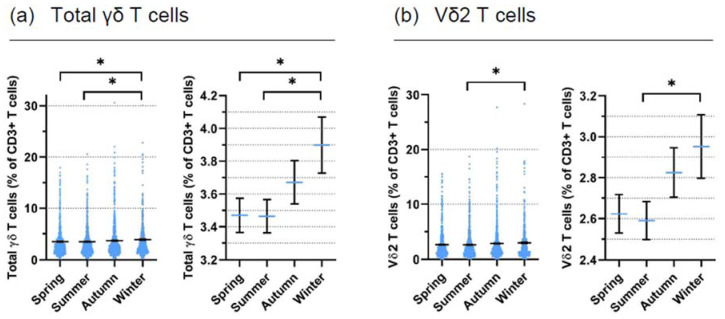
**Seasonal variation of γδ T cells in random blood donors over ten years**. A total of 2625 blood samples from random healthy adult blood donors collected from 2011 to 2020 were analyzed for the proportion of total γδ T cells (**a**) and Vδ2 T cells (**b**) among CD3^+^ T cells. In the left panels, all individual data points are displayed, while in the right panels, mean values ± SEM are shown. For statistical analysis, a linear regression was performed. The dependent variable was total γδ T cells (R^2^ = 0.003) in (**a**) and Vδ2 T cells (R^2^ = 0.002) in (**b**). For statistical comparison, the reference season was Winter, * *p* < 0.05.

Next, we combined results from individual months into four seasons (Spring: March, April, May; Summer: June, July, August; Autumn: September, October, November; Winter: December, January, February). The results of the ten years from all donors according to the season are displayed for total γδ T cells in Figure 1a and for Vδ2 T cells in Figure 1b. In the left panels, all data points and the means are displayed to illustrate the range of γδ T cell proportions, while in the right panels, mean values ± SEM with a higher resolution on the *y* axis are displayed to better visualize seasonal differences. Significantly higher total γδ and Vδ2 T cell proportions were observed in winter when compared with summer, and in the case of total γδ T cells also when compared with spring. The screening of random blood donors over 10 years was performed because of our continued interest in identifying donors with reasonable proportions of γδ T cells for a variety of functional experiments with isolated γδ T cells. For this reason, we did not include other markers such as CD4 and CD4 in the screening, which would have provided important additional information on the seasonal variation of immune cells. 

While many parameters might influence the seasonal fluctuation of immune cell frequencies, one such parameter could be vitamin D_3_ which is subject to seasonal alterations related to the intensity of sunshine exposure. Furthermore, cholesterol, which is the precursor molecule of vitamin D synthesis, is a metabolite of the mevalonate pathway, which generates Vγ9Vδ2 T cell-activating pyrophosphates (IPP, geranyl pyrophosphate) [8]. Therefore, our next step was to perform a prospective immunophenotypic study with up to eight time points over one year in a small cohort of healthy adult donors who did or did not take oral vitamin D_3_ supplementation. 

### 3.2. Prospective Analysis

Characteristics of blood donors are summarized in Supplementary Table S1. There were 14 men and 17 women; the mean age was 27.3 ± 3.3 years. Among them, 14 donors did not take any oral vitamin D_3_ (group 1), 9 donors took vitamin D_3_ regularly throughout the year (group 3), while 8 donors took vitamin D_3_ irregularly (group 2). The gating strategy for measuring total γδ T cells and Vδ2 T cells among CD3^+^ T cells is shown in Supplementary Figure S3.

**Figure 2:**
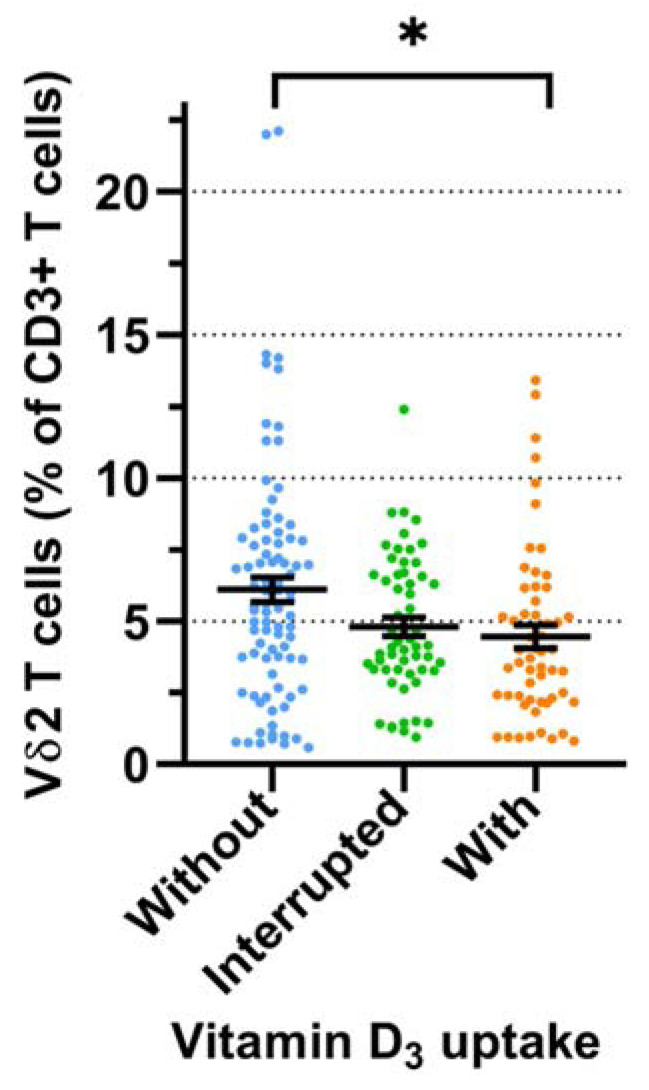
**The proportion of Vδ2 T cells among CD3^+^ T cells according to oral vitamin D_3_ supplementation.** All analyzed time points of all donors in three groups are displayed: group 1 (blue): no oral vitamin D_3_, 14 donors; group 2 (green): interrupted oral vitamin D_3_ supplementation, 8 donors; group 3 (orange): continuous vitamin D_3_ uptake, 9 donors. Bars represent mean values ± SEM. Statistical comparison was made by Kruskal–Wallis test and Dunn’s multiple comparison test. * *p* = 0.0113.

We first compared Vδ2 T cell proportions according to oral vitamin D_3_ uptake in all respective donors per group and across all time points (Figure 2).

**Figure 3:**
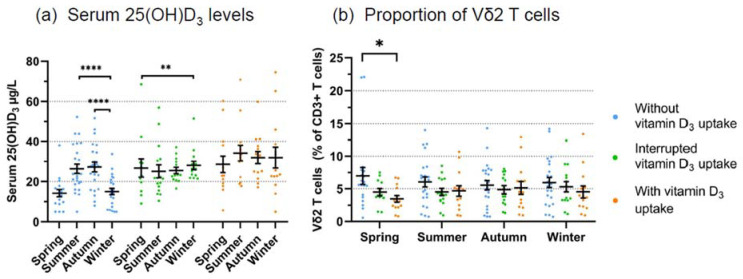
**Seasonal fluctuation of serum 25(OH)D_3_ levels and proportion of Vδ2 T cells according to oral vitamin D_3_ supplementation.** (**a**) Serum levels of 25(OH)D_3_ in donors without vitamin D_3_ supplementation (left panel, blue symbols), in donors with interrupted vitamin D_3_ uptake (middle panel, green symbols), and in donors with regular vitamin D_3_ uptake (right panel, orange symbols). Statistical comparison was made by Wilcoxon matched pairs signed-rank test, by testing each season against winter. (**b**) The proportion of Vδ2 T cells among CD3 T cells according to the season in the same individuals. Bars represent mean values ± SEM. Statistical comparison was made by Kruskal–Wallis test and Dunn’s multiple comparison test. * *p* = 0.0276; ** *p* < 0.01, and **** *p* < 0.0001.

In this analysis, the donors who did not take vitamin D_3_ (group 1) had higher Vδ2 T cell proportions compared with the donors who regularly took vitamin D_3_ (group 3) (mean 6.1% vs. 4.5%, *p* < 0.05). Next, we determined serum levels of 25(OH)D_3_ and the proportion of Vδ2 T cells in the three groups according to the seasons (defined as in Section 3.1). Donors who did not take oral vitamin D_3_ (group 1) had low serum levels (<20 µg/mL) of 25(OH)D_3_ in spring and winter and—as expected—significantly higher levels in summer and autumn due to increased sun exposure (Figure 3a, left panel). Donors who took vitamin D_3_ with an interruption during the summer months (group 2; Figure 3a, middle panel) or regularly throughout the year (group 3; Figure 3a, right panel) had, on average, normal (>20 µg/mL) serum levels of 25(OH)D_3_ throughout the year.

We then determined the proportion of Vδ2 T cells among CD3 T cells in the three groups and according to the season. Throughout the year, the highest mean values for Vδ2 T cell proportions were always observed in group 1 donors without oral vitamin D_3_ supplementation (Figure 3b). This tendency was most prominent in spring when the mean Vδ2 T cell proportion was 7.0% in group 1, 4.5% in group 2, and 3.5% in group 3 donors (*p* = 0.0276). However, there was no direct correlation between the actually measured 25(OH)D_3_ serum levels with the proportion of Vδ2 T cells across all donors (Supplementary Figure S4). In addition to Vδ2 T cells which dominate in peripheral blood, we also analyzed seasonal changes in the proportion of the minor subset of non-Vδ2 γδ T cells (which mainly comprises Vδ1 γδ T cells), but there was no obvious seasonal fluctuation in non-Vδ2 γδ T cells (Supplementary Figure S5a). The proportion of non-Vδ2 γδ T cells also did neither correlate with low (<20 µg/L) or higher serum levels of 25(OH)D_3_ (Supplementary Figure S5b) nor with oral vitamin D_3_ (Supplementation (Supplementary Figure S5c)).

**Figure 4:**
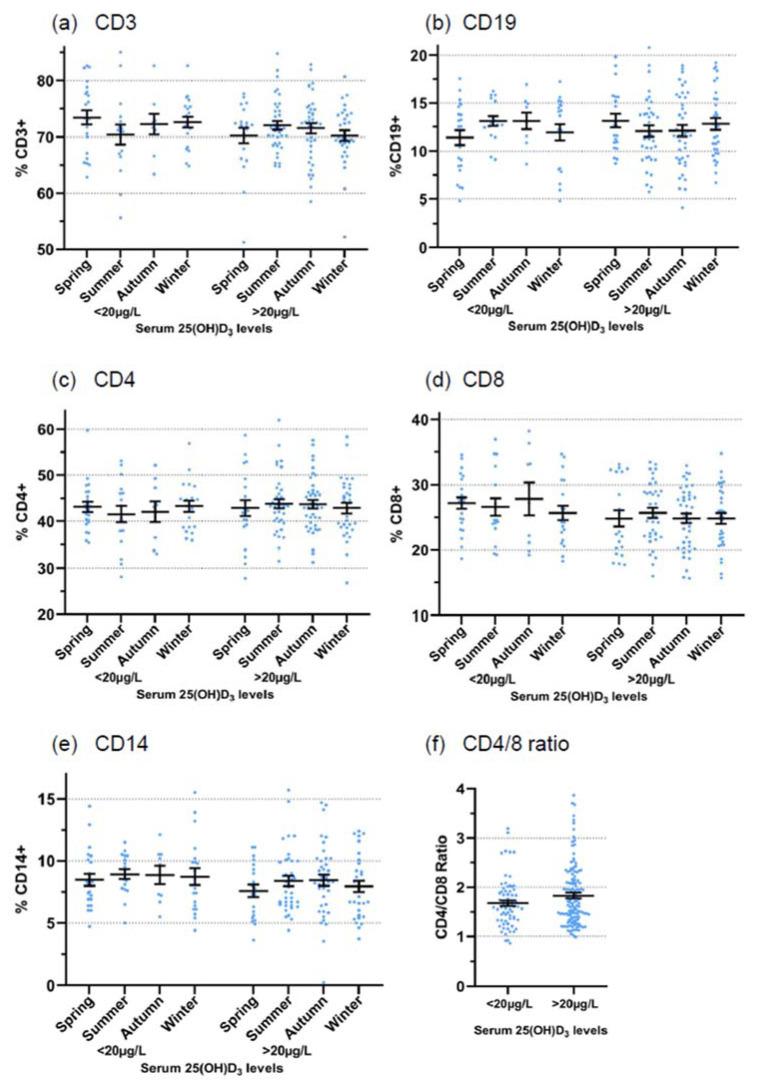
**Seasonal variation of immune cell subsets and correlation with serum 25(OH)D_3_ levels.** Proportions of CD3 (**a**), CD19 (**b**), CD4 (**c**), CD8 (**d**), and CD14 (**e**) cells were measured in blood samples from all donors in the prospective study. Mean values ± SEM and individual data points are displayed according to the season and according to the serum level of 25(OH)D_3_: low (left panels: <20 µg/L), normal (right panels: >20 µg/L). (**f**) CD4/CD8 ratio according to serum 25(OH)D_3_ levels (all donors, all time points). Statistical comparison in (f) was made by Mann–Whitney *U* test but did not reach significance.

In addition to γδ T cells, we also performed immunophenotyping of conventional immune cell subsets (CD3, CD4, CD8, CD19, CD14) in the prospective study. To delineate a possible influence of serum 25(OH)D_3_ levels, we grouped all study participants into low (<20 µg/L) and normal (>20 µg/L) serum levels and according to the season. These results are presented in Figure 4a–e. While there was a slight tendency for lower CD8 T cell proportions when serum levels of 25(OH)D_3_ were > 20 µg/L (Figure 4d), no clear-cut correlation between 25(OH)D_3_ serum levels and immune cell subset distribution emerged. Relative proportions of immune cell subsets are shown in Figure 4, but similar patterns were observed if absolute cell counts were considered (results not shown). Because of the slightly reduced CD8 T cell proportions (Figure 4d), a slightly higher CD4/CD8 ratio was observed in donors with higher 25(OH)D_3_ serum levels (Figure 4f; mean 1.83 vs. 1.67, statistically not significant). For comparison, we also performed a similar analysis for total γδ T cells and Vδ2 T cells (Supplementary Figure S6a,b).

### 3.3. Modulation of γδ T Cell Activation In Vitro

In the third part of this study, we investigated the modulatory role of vitamin D_3_ on the in vitro activation of γδ T cells. To this end, we investigated the effects of the active vitamin D_3_ metabolite 1α,25-Dihydroxyvitamin D_3_ [1,25(OH)_2_D_3_] on the proliferative activity, antitumor cytotoxicity, and cytokine production of γδ T cells.

#### 3.3.1. Inhibition of γδ T Cell Expansion

**Figure 5:**
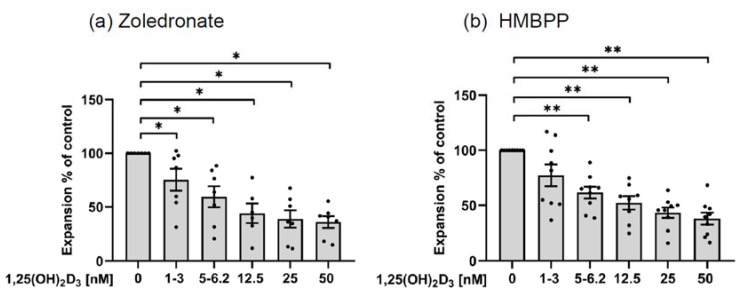
**1,25(OH)_2_D_3_ inhibits γδT cell expansion**. PBMC from healthy donors were activated with 2.5 µM Zoledronate (**a**) or 10 nM HMBPP (**b**) in the presence of IL-2 and the indicated concentrations of 1,25(OH)_2_D_3_. After 8 d, the absolute number of viable Vγ9 T cells was determined. The number of viable γδ T cells in control cultures without 1,25(OH)_2_D_3_ was set as 100%, and the relative growth of γδ T cells in the presence of 1,25(OH)_2_D_3_ was calculated. Mean ± SEM of 8–9 (**a**) and 6–7 (**b**) independent experiments are shown. Statistical significance was analyzed by the Wilcoxon matched pairs signed-rank test. * *p* < 0.05, ** *p* < 0.01.

PBMC were activated with predetermined optimal concentrations of ZOL (2.5 µM) or HMBPP (10 nM) in the presence of 50 IU/mL IL-2 and titrated concentrations of 1,25(OH)_2_D_3_, or solvent control DMSO corresponding to the highest possible concentration in which 1,25(OH)_2_D_3_ was dissolved (1:1000). Dose-dependent inhibition of T cell proliferation was observed macroscopically after 6 d (Supplementary Figure S7a) and could be verified upon microscopic inspection (Supplementary Figure S7b). Next, we quantified the inhibitory effect of 1,25(OH)_2_D_3_ on γδ T cell proliferative expansion stimulated by ZOL or HMBPP. To this end, PBMC were cultured in 96-well round-bottom plates and were activated by 2.5 µM ZOL or 10 nM HMBPP in the presence of IL-2 and titrated concentrations of 1,25(OH)_2_D_3_. After 8 d, the absolute number of viable Vγ9 T cells per microculture well was determined, and the measured cell number in untreated control cultures was set as 100%. As shown for ZOL in Figure 5a and HMBPP in Figure 5b, 1,25(OH)_2_D_3_ dose-dependently inhibited the Vγ9 T cell expansion to both selective γδ T cell stimuli, with >50% inhibition observed at 50 nM 1,25(OH)_2_D_3_. The solvent control DMSO at 1:1000 dilution did not affect γδ T cell expansion (not shown). In the SCDA analysis, cell cultures are stained with propidium iodide (PI) to exclude dead cells (see Materials and Methods). While there was more cell death of Vγ9 T cells (i.e., PI^+^Vγ9^+^) in ZOL-stimulated cultures in comparison with HMBPP-activated cell cultures, this was not modulated in the presence of 1,25(OH)_2_D_3,_ suggesting induction of apoptosis was not the main reason for the observed growth inhibition (Supplementary Figure S8).

#### 3.3.2. Modulation of Cytotoxic Effector Activity

**Figure 6:**
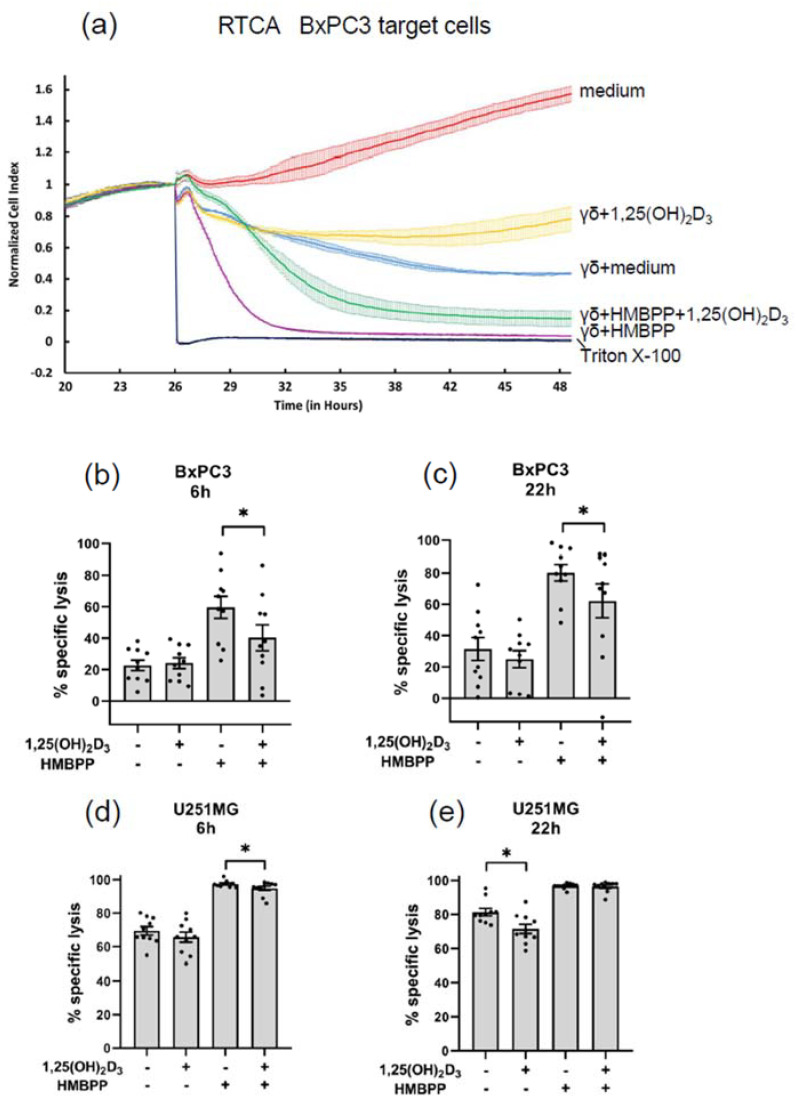
**Cytotoxic activity of γδ T cells expanded in the absence or presence of 1,25(OH)_2_D_3_**. Vδ2 T cell lines expanded for 14 d from ZOL-stimulated PBMC in the absence or presence of 50 nM 1,25(OH)_2_D_3_ were used as effector cells (E/T ratio 10:1) in the RTCA with BxPC3 (**a**–**c**) or U251MG target cells (**d**,**e**). Where indicated, HMBPP was added at a final concentration of 10 nM. The impedance was continuously recorded over 48 h beginning with the addition of tumor cells. Effector cells were added at time point 26 h, and the % specific lysis was calculated in relation to spontaneous tumor cell growth and Triton X-100 induced maximal lysis at 6 h and 22 h after addition of effector cells, based on the normalized cell index, which was set to 1 at the time when effector cells were added. (**a**) RTCA plot with one Vδ2 effector cell population and BxPC3 tumor target cells. (**b**,**c**) Specific lysis of BxPC3 target cells at 6 h (**b**) and 22 h (**c**) by control or 1,25(OH)_2_D_3_-expanded Vδ2 effector T cells in the absence or presence of HMBPP. (**d**,**e**) Specific lysis of U251MG target cells at 6 h (**d**) and 22 h (**e**) by control or 1,25(OH)_2_D_3_-expanded Vδ2 effector T cells in the absence or presence of HMBPP. Mean ± SEM of experiments with 10 different Vδ2 T cell lines is shown. Statistical significance was analyzed by the Wilcoxon matched pairs signed-rank test. * *p* < 0.05.

Short-term γδ T cell lines to be used as effector cells in cytotoxicity assay were generated by activating PBMC with ZOL and IL-2 in the absence or presence of 50 nM 1,25(OH)_2_D_3_. Cell cultures were supplemented with IL-2 every two days and were split when appropriate. 1,25(OH)_2_D_3_ was added once again after 7 d. Such cell lines contained 74–92% CD3^+^ Vδ2^+^ γδ T cells after 14 d, the time point when cells were washed and used as effector cells in RTCA cytotoxicity assays with pancreatic adenocarcinoma BxPC3 or glioblastoma U251MG target cells. In some experiments, γδ T cells were used earlier than d 14. The RTCA plot over a total time period of 48 h of one experiment with BxPC3 target cells is shown in Figure 6a. Tumor cells were seeded at time point 0 h, and effector cells +/− HMBPP, as well as Triton-X 100, were added at time point 26 h. From this time point, the RTCA continued for another 22 h. Cytotoxicity after 6 h of coculture thus corresponds to time point 32 h in Figure 6a, whereas cytotoxicity after 22 h corresponds to time point 48 h in this graph. As expected, the addition of the TCR stimulus HMBPP to the assay greatly increased the cytotoxic activity of expanded Vδ2 T cells. Interestingly, the Vδ2 effector T cells expanded in the presence of 1,25(OH)_2_D_3_ were less active in killing BxPC3 target cells, as evidenced by the yellow line (1,25(OH)_2_D_3_) in comparison with the blue line (medium) in Figure 6a. The lower activity of 1,25(OH)_2_D_3_-expanded Vδ2 effector T cells was also not fully restored in the presence of HMBPP (compare purple and green lines).

A summary of experiments with 10 different Vδ2 T cell lines generated from different donors and BxPC3 target cells at an E/T ratio of 10:1 is shown in Figure 6b (time point 6 h) and Figure 6c (time point 22 h). Lysis of BxPC3 cells in the absence of HMBPP was moderate after 6 and 22 h and tended to be lower at 22 h with Vδ2 effector cells generated in the presence of 1,25(OH)_2_D_3_, which, however, did not reach statistical significance. As expected, lysis at both 6 and 22 h was strongly increased in the presence of HMBPP. Interestingly, at both time points, killing by 1,25(OH)_2_D_3_-expanded Vδ2 effector cells in the presence of HMBPP was slightly but significantly reduced (*p* < 0.05).

U251MG glioblastoma target cells are known to be more sensitive to γδ T cell lysis compared with BxPC3 [35]. In line, the strong killing of U251MG target cells by ZOL-expanded γδ T cells was already observed after 6 h (Figure 6d) and was further increased after 22 h (Figure 6e). When measured after 22 h, Vδ2 T cells expanded in presence of 1,25(OH)_2_D_3_ were slightly but significantly less efficient in killing U251MG tumor cells (Figure 6e). The addition of HMBPP further enhanced the killing activity, both at 6 h and 22 h. In the presence of HMBPP, there was almost complete lysis already after 6 h, and this was slightly reduced with Vδ2 effector T cells expanded in the presence of 1,25(OH)_2_D_3_. 

#### 3.3.3. Modulation of Cytokine Production

**Figure 7:**
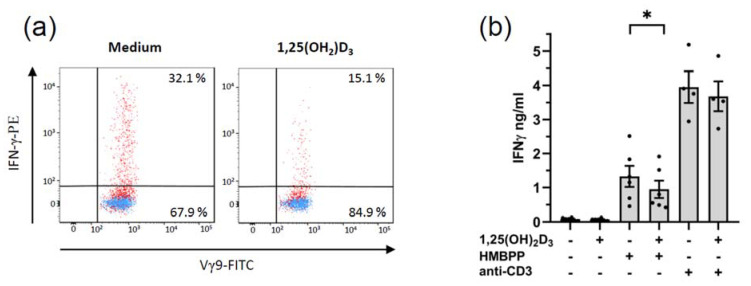
**1,25(OH)_2_D_3_ inhibits IFN-γ induction in γδ T cells.** (**a**) PBMC were activated with 2.5 µM ZOL in the absence (left dot plot) or presence (right dot plot) of 50 nM 1,25(OH)_2_D_3_. After 48 h, cells were stained for intracellular detection of IFN-γ in CD3^+^Vγ9^+^ T cells. One of two experiments is shown. (**b**) γδ T cell lines generated from ZOL-activated PBMC and expanded for 14 d were cultured overnight in a medium (*n* = 6), with HMBPP (*n* = 6) or immobilized anti-CD3 antibody (*n* = 4) in the absence or presence of 50 nM 1,25(OH)_2_D_3_, as indicated. IFN-γ in culture supernatants was measured by ELISA. Statistical analysis was performed with Wilcoxon matched pairs signed-rank test. * *p* = 0.0313.

Finally, we analyzed the effect of 1,25(OH)_2_D_3_ on the IFN-γ production by activated γδ T cells in two different setups, namely by flow cytometry in γδ T cells within freshly isolated PBMC and by ELISA in supernatants of expanded γδ T cell lines. To this end, PBMC were activated with ZOL, and short-term expanded γδ T cell lines were restimulated with HMBPP or immobilized anti-CD3 antibody. As shown in Figure 7a (left dot plot), 32.1% of γδ T cells stained positive for intracellular IFN-γ when PBMC were activated for 48 h with ZOL. In the presence of 1,25(OH)_2_D_3_, this proportion was reduced to 15.1% (Figure 7a, right dot plot). γδ T cell lines expanded for 14 d secreted IFN-γ when restimulated overnight with HMBPP or with immobilized anti-CD3 antibody (Figure 7b). The presence of 1,25(OH)_2_D_3_ significantly reduced the IFN-γ secretion in response to HMBPP (mean 0.95 ng/mL vs. 1.33 ng/mL). Stimulation with immobilized anti-CD3 antibody is a much stronger activation signal and induced a threefold higher concentration of secreted IFN-γ (mean 3.95 ng/mL). Again, this was reduced by 1,25(OH)_2_D_3_ (mean 3.67 ng/mL), but the inhibition did not reach statistical significance (Figure 7b). 

## 4. Discussion

Under physiologic conditions, the homeostasis of the immune system is well controlled. While the global immune cell composition is influenced by genetic background, sex, age, and environmental factors such as a persistent CMV infection [36,37,38], there are also variations over time at the individual level. These include seasonal variability in gene expression and some immune parameters, but also nonseasonal longitudinal variation in functional immune responses [39,40,41]. Previous studies have investigated the fluctuation of defined immune cell subsets in healthy individuals at several time points during one year, thus providing a snapshot of variation during the seasons. Using multicolor flow cytometry, Khoo et al. [27] observed enhanced CD4, and to a lesser extent CD8, T cell counts in spring and summer compared with winter, as well as seasonal variation in homing marker expression on CD4 T cells. Only moderate variability over time in relation to the season was reported in more recent studies employing multicolor flow cytometry or high throughput mass cytometry for phenotyping [40,41]. Interestingly, CD4^+^CD25^high^ regulatory T cells (Treg) were found to be most strongly affected by seasonality, with their frequency peaking in autumn [40]. However, the minor population of γδ T cells was not included in previous analyses [27,40,41].

Many factors might impact the variation of immune parameters, including the contrasting weather conditions in summer vs. winter, but also physical activity and diet. Exposure to solar or artificial ultraviolet radiation has an impact on immune cells in human blood [42]. In this context, vitamin D is an important factor that is not only a regulator of calcium and phosphate metabolism but also a potent modulator of immune cell function [18,21]. The essential step is the generation of vitamin D_3,_ which results from the absorption of UV-B radiation (and thus sun exposure) by 7-dehydrocholesterol in the skin. The subsequent metabolism in the liver and kidney produces the biologically active 1,25(OH)_2_D_3,_ which exerts its functional activity via the nuclear vitamin D receptor (VDR). Vitamin D sensing by the transcription factor VDR initiates epigenetic and transcriptional regulation of a plethora of target genes [43]. Serum levels of 25(OH)D_3_ (the immediate precursor of 1,25(OH)_2_D_3_) vary considerably, with higher levels measured in summer when sun exposure is more intensive compared with winter [44]. In view of its immunoregulatory role, vitamin D3 deficiency has been associated with various diseases, notably with the onset of various autoimmune diseases and the incidence and severity of infections, including upper respiratory tract infections caused by influenza and SARS-CoV-2 [17,18,45]. To maintain sufficient serum levels of vitamin D_3_ throughout the year, the oral supplementation with vitamin D_3_ (cholecalciferol) or (less frequently) vitamin D_2_ (ergocalciferol) is widely recommended, even though there is no uniform opinion about the optimal dosage and serum concentration [18,45,46,47].

The immunomodulatory effects of vitamin D_3_ might be beneficial on the basis of several mechanisms of action which have been described. It must be emphasized, however, that there is also controversy as to how some of the in vitro observations translate into measurable effects in vivo upon oral vitamin D_3_ uptake [48]. This notwithstanding, it is obvious that vitamin D_3_ affects both innate and adaptive immunity. The vitamin D receptor (VDR) has been shown to act as a negative regulator of the NLRP3 oligomerization and activation. In consequence, vitamin D3 inhibits NLRP3 inflammasome activation and IL-1β secretion and thus might be useful in the treatment of inflammatory conditions [49]. At the crossroads between innate and adaptive immunity, vitamin D3 induces a tolerogenic phenotype in dendritic cells (DCs) [50], which has recently been linked to JAK2-mediated STAT3 phosphorylation [51]. The induction of tolerogenic DCs might help control autoreactive T cells in autoimmune diseases. This could also be supported by the promotion of Treg activation and suppressive activity by vitamin D3. In several studies, vitamin D3 was found to increase the expression of the Treg-specific transcription factor FoxP3 in naturally occurring Treg [20,52,53,54], and this may be associated with enhanced suppressive activity, cell cycle progression, and proliferation of Treg [54,55,56,57]. However, additional mechanisms could contribute to the potentially beneficial effect of vitamin D3 on autoreactive T cells, including the upregulation of immunosuppressive CD73, the induction of IL-10, and the inhibition of proinflammatory Th17 cells [20,58,59,60,61].

The focus of our study was twofold, i.e., (i) to investigate the seasonal variation of γδ T cells in healthy adult individuals and (ii) to analyze the possible impact of vitamin D_3_ on the seasonal frequency and on in vitro activation of γδ T cells. Even though a numerically small subset of peripheral blood T cells, γδ T cells fulfill important functions in the immune response to infection and stressed/transformed cells [10,11,12]. Because of their HLA-nonrestricted mode of action and potent cytotoxic activity, multiple approaches are currently under investigation to apply γδ T cells in immunotherapy of cancer and viral (re)infection [10,11,12,62,63]. The recent demonstration that it is safe to transfer γδ T cells expanded in vitro from healthy donors across HLA barriers into cancer patients has opened the way to apply off-the-shelf γδ T cell therapy to treat cancer patients [64].

Our retrospective analysis of 2625 samples of random blood donors over ten years revealed small but statistically significant differences in the proportion of γδ T cells (both total γδ T cells and the Vδ2 subset) across the seasons, with higher γδ T cell proportions recorded in winter compared with spring and summer. We are fully aware of the limitations of this analysis as we did not consider confounders such as age, sex, potential oral vitamin D_3_ supplementation, etc. We performed the screening over 10 years to identify random blood donors with sufficient numbers of γδ T cells for various γδ T cell-focused projects. It is certainly an additional limitation of the retrospective analysis that we did not include other markers such as CD4 and CD8. Nevertheless, in view of the very large sample number, we took this as an indication that there might be a seasonal variation of γδ T cells circulating in the blood. Furthermore, we considered that this could be influenced by vitamin D_3_ levels, which are higher in summer compared with winter. Given the paucity of the information in the literature, this is the first hint for seasonal variation of γδ T cells in a large cohort of random healthy adult blood donors. To investigate the seasonal fluctuation in individual donors, we initiated a small prospective study with 31 healthy adults who did or did not take oral vitamin D_3_ supplementation. We performed immunophenotyping, including γδ T cells and conventional immune cell subsets at up to eight time points over one year; serum levels of 25(OH)D_3_ were measured at the same time points. Several observations emerged from this analysis which point to a possible influence of vitamin D_3_ on circulating γδ T cell frequency. First, the analysis of all data points from all donors indicated a higher proportion of Vδ2 T cells in donors who did not take oral vitamin D_3_ in comparison with individuals who took vitamin D_3_ supplementation throughout the year (Figure 2). Secondly, the measured serum levels of 25(OH)D_3_ correlated as expected with the practice of taking oral vitamin D_3_ or not. Thus, low levels were measured in spring and winter, and higher levels in summer and autumn in individuals who did not take any oral vitamin D_3_. By contrast, higher levels with little seasonal variation were measured both in individuals who regularly took vitamin D_3_ and in those who took vitamin D_3_ with interruptions during the summer (Figure 3a). In line with the assumption that low levels of vitamin D_3_ are associated with higher proportions of circulating γδ T cells, we measured, on average, the highest γδ T cell frequencies across the four seasons in donors without oral vitamin D_3_ supplementation, whereas lower γδ T cell proportions were detected during the year in individuals taking oral vitamin D_3_. This difference reached statistical significance in spring but not in the other seasons (Figure 3b). These results suggest that vitamin D_3_ might have a negative impact on the proportion of circulating γδ T cells, an assumption which would be in line with the observed suppressive effects of the active vitamin D_3_ metabolite 1,25(OH)_2_D_3_ in vitro (see below). Obviously, a verification of this hypothesis will require a larger prospective study with more individuals and under strictly controlled vitamin D_3_ uptake conditions. In a previous study on osteoporosis patients who were on ZOL treatment, De Santis et al. did not observe a correlation between 25(OH)D3 serum levels and γδ T cell numbers [65]. However, this study cannot be directly compared with our results in untreated healthy individuals since the therapeutic application of ZOL induces in vivo activation of γδ T cells but also leads to a depletion of circulating γδ T cells upon prolonged treatment with aminobisphosphonates [66,67]. In parallel to γδ T cells, we also analyzed the frequency of other immune cells in the prospective cohort and categorized the results according to the measured 25(OH)D_3_ serum levels. The proportion of CD8 T cells tended to be lower in individuals with higher serum levels of 25(OH)D_3_, resulting in a slightly elevated CD4/CD8 ratio as compared with individuals with <20 μg/L 25(OH)D_3_. Overall, these results of immunophenotyping are in line with previous studies with more extensive marker panels [40,41]. However, it is well possible that serum 25(OH)D_3_ levels and vitamin D_3_ uptake have more pronounced effects on particular subsets of conventional CD4 and CD8 T cells. In this respect, Khoo et al. observed seasonal variation of CD4 T cell subsets defined by memory marker and homing receptor expression [27].

Seasonal variation in the intensity of UV radiation may also affect other factors relevant to the immune system, such as nitric oxide (NO). NO is an important modulator of T cell activation [68,69] and is generated in the skin by UV-A and near-infrared wavelength [70,71]. It is currently unknown how skin-derived NO might affect γδ T cell plasticity in vivo.

We also investigated the modulation of in vitro activation and effector functions of γδ T cells by the active metabolite 1,25(OH)_2_D_3_. Numerous previous studies have reported the modulation of CD4 T cell differentiation by 1,25(OH)_2_D_3_, but only one previous study has focused on γδ T cells [28]. The accumulated evidence supports the notion that vitamin D_3_ inhibits CD4 T cell proliferation and inhibits their inflammatory gene program by suppressing IL-17 and IL-9 and favoring IL-10 induction [19,72,73,74,75]. A recent molecular analysis demonstrated that Th1 cells could turn off their proinflammatory cytokine program in an autocrine manner and switch to IL-10 production in response to vitamin D_3_, a process that depends on several transcription factors, including c-JUN, STAT3, and BACH2 [23]. We observed that 1,25(OH)_2_D_3_ drastically inhibited the in vitro expansion of γδ T cells when PBMC were stimulated with γδ T cell-specific ligands such as ZOL and HMBPP. The experiments were carried out in the presence of exogenous IL-2, so growth inhibition did not occur at the level of endogenous IL-2 production. Using a different read-out system, Chen and colleagues also reported γδ T cell growth inhibition by 1,25(OH)_2_D_3_ [28]. The activation of γδ T cells within PBMC using ZOL as a stimulus completely depends on the presence of monocytes [8]. One possible reason for the inhibition seen in the presence of 1,25(OH)_2_D_3_ could be that 1,25(OH)_2_D_3_ interferes with the production of the γδ T cell-activating pAg IPP in monocytes which would have to be analyzed in future studies. On the other hand, proliferative expansion was also inhibited in response to the synthetic pAg HMBPP, which is less dependent on monocytes but also requires accessory cells [76]. Our data extracted from the SCDA experiments do not suggest that there was significant additional cell death of γδ T cells in the presence of up to 50 nM 1,25(OH)_2_D_3_. This indicates that growth inhibition might result from cell cycle arrest rather than induction of apoptosis. On the other hand, Chen et al. observed increased cell death when γδ T cells were activated with IPP and higher (100 nM) concentrations of 1,25(OH)_2_D_3_ than used in our study [28]. Taken together, the analysis of the precise molecular mechanisms of growth inhibition of γδ T cells by 1,25(OH)_2_D_3_ will require further investigation.

We also observed that 1,25(OH)_2_D_3_ inhibits the IFN-γ production in human γδ T cells. Inhibition of IFN-γ induction in γδ T cells within PBMC was previously reported by Chen et al. [28], and we confirmed this observation in a slightly different system. Again, we stimulated PBMC with ZOL in the absence or presence of 1,25(OH)_2_D_3_. When analyzed after 48 h, the proportion of γδ T cells expressing IFN-γ was markedly reduced in the presence of 1,25(OH)_2_D_3_. Under these conditions, it is likely that monocytes play an essential role. We extended these experiments to demonstrate that 1,25(OH)_2_D_3_ also reduced IFN-γ secretion when expanded γδ T cell lines are stimulated with pAg or anti-CD3 antibody. While the effect in the anti-CD3 antibody stimulation was minimal, it was significant when γδ T cell lines were activated with HMBPP. Therefore, 1,25(OH)_2_D_3_ can directly act on activated γδ T cells, in line with the demonstration that activated T cells express a functional VDR [28,77]. While our and the previous results [28] clearly demonstrate that 1,25(OH)_2_D_3_ suppresses IFN-γ induction in human γδ T cells, it remains to be investigated if 1,25(OH)_2_D_3_ might also act to drive γδ T cell differentiation towards IL-10 production.

γδ T cell lines expanded in the presence of 1,25(OH)2D3 displayed reduced cytotoxic activity towards two different tumor target cells. The RTCA system applied here has been extensively used to follow the cytotoxic effect of γδ T cells on tumors over extended time periods [34,35]. The two tumor cell lines applied here are differentially susceptible to γδ T cell-mediated lysis. The glioblastoma U251MG is highly susceptible, while the pancreatic adenocarcinoma BxPC3 is much less susceptible [35]. In all settings, 1,25(OH)_2_D_3_-expanded γδ T cells were not more active than the control γδ T cell lines. In fact, the enhanced lysis of BxPC3 target cells noticed in the presence of the TCR-activating pAg HMBPP was also reduced at both early (6 h) and later (22 h) time points by 1,25(OH)_2_D_3_ treatment. There were fewer differences between control and 1,25(OH)_2_D_3_-expanded γδ T cells when U251MG were used as target cells, but again, there was some reduction in the absence of HMBPP after 22 h and a minor reduction in the presence of HMBPP after 6 h. Again, the underlying mechanisms have not been investigated in more detail in this study. γδ T cells can utilize both death receptor/ligand pathways (e.g., Fas/CD95, TRAIL) as well as the secretory pathway (perforin, granzyme B, granulysin) to kill target cells [78,79,80]. Previous investigations have demonstrated that 1,25(OH)_2_D_3_ downregulates the cytotoxic effector response in pulmonary tuberculosis by reducing the expression of perforin, granulysin, and granzyme B [81]. It remains to be investigated if a similar mechanism contributes to the regulation of cytotoxic function in human γδ T cells by vitamin D_3_.

While binding of 1,25(OH)_2_D_3_ to the nuclear VDR is an established mode of action, it is also known that several other vitamin D_3_ hydroxyderivatives can act on other nuclear receptors such as the liver X receptors (LXRs) and retinoic acid-related orphan receptors (RORs) [82,83]. How the activation of such receptors by vitamin D_3_ hydroxyderivatives might impact the in vivo fluctuation of immune cells and the in vitro activation of γδ T cells is presently unknown.

Overall, the results of our in vitro studies imply that 1,25(OH)_2_D_3_ exerts mainly inhibitory effects on human γδ T cells (at least in the read-out systems analyzed here). If relevant in vivo, such an inhibitory effect could be in line with the higher proportion of γδ T cells in the blood of individuals with low 25(OH)D_3_ serum levels in spring and winter—a hypothesis that certainly needs further validation.

The results of our study are of interest in the context of the potential application of exogenous vitamin D_3_ in diseases, notably when harnessing γδ T cells for immunotherapy of cancer. Enhancing Treg activity and modulating T cell differentiation by suppressing Th1 and Th17 responses towards IL-10 induction by vitamin D_3_ supplementation might be advisable for autoimmune and inflammatory diseases [84]. However, the inhibition of effector functions might also have a negative impact on T cell-mediated antitumor immunity, as recently shown in a 3D tumor spheroid model [85]. This notwithstanding, vitamin D_3_ has been proposed to exert anticancer effects by acting on the tumor microenvironment as well as directly on some tumor cell types [86,87]. Given the current enthusiasm to translate the unique features of γδ T cells into novel cancer immunotherapies, further studies are warranted to investigate in more detail the impact of vitamin D_3_ and its therapeutic application on the γδ T cell compartment. 

## 5. Conclusions

Our study points to a possible role of vitamin D_3_ in the homeostasis of peripheral blood γδ T cells, which needs to be verified in a larger cohort and under well-controlled conditions of vitamin D_3_ supplementation. In vitro, the active vitamin D_3_ metabolite 1,25(OH)_2_D_3_ inhibited γδ T cell activation and effector functions. Oral vitamin D3 supplementation is frequently recommended to cancer patients, and γδ T cells are in development as novel cellular cancer immunotherapy. Our study raises a *caveat* as to whether (high) dose vitamin D_3_ therapy is advisable during γδ T cell immunotherapy by adoptive γδ T cell transfer of in vivo activation of γδ T cells [12].

## Data Availability

The data underlying the results presented in this paper are available upon reasonable request.

## Supplementary Materials

The following supporting information can be downloaded at: https://www.mdpi.com/article/10.3390/cells11091460/s1, Table S1: characteristics of 31 prospective study group participants, Figure S1: gating strategy for retrospective analysis, Figure S2: mean values of total γδ T cells and Vδ2 T cells in retrospective analysis, Figure S3: gating strategy for prospective analysis, Figure S4: correlation of Vδ2 T cells with serum 25(OH)D_3_ levels, Figure S5: seasonal analysis of non-Vδ2 γδ T cells and correlation with Vitamin D_3_ in the prospective analysis, Figure S6: seasonal variation of γδ T cells and correlation with serum 25(OH)D_3_ levels, Figure S7: growth inhibition of Zoledronate-activated PBMC by 1α,25(OH)_2_D_3_. Figure S8: influence of 1α,25(OH)_2_D_3_ on cell death of Vγ9 T cells.
