# Supplementary material for: Analysis of the Seasonal Fluctuation of γδ T Cells and Its Potential Relation with Vitamin D3
Source: Cells. 2022 Apr 26;11(9):1460. doi: 10.3390/cells11091460 (PMC9099506; doi:10.3390/cells11091460)
Supplement: Supplementary file 1 [file cells-11-01460-s001.zip › cells-1664613-supplementary.pdf]

Supplemental Material

Analysis of the seasonal fluctuation of γδ T cells and its potential relation with Vitamin D_3

Birthe Bernicke, Nils Engelbogen, Katharina Klein, Jeanette Franzenburg, Christoph Borzikowsky, Christian Peters, Ottmar Janssen, Ralf Junker, Ruben Serrano and Dieter Kabelitz

Supplemental Table 1:

Characteristics of 31 prospective study group participants

**Gender:**	14 males<br>17 females
**Age:**	mean 27.3 ± 3.3 years, range 22 to 35 years

Oral Vitamin D uptake*:

Group 1 (none):	14 (6 males, 8 females)
Group 2 (interrupted during summer):	8
Group 3 (regular throughout year):	9

* Vitamin D uptake: minimum 500 I.E. per day, maximum 20,000 I.E. per day, mean 1,877 ± 1,968 I.E. per day

Supplemental Figure S1:

Gating strategy for retrospective analysis

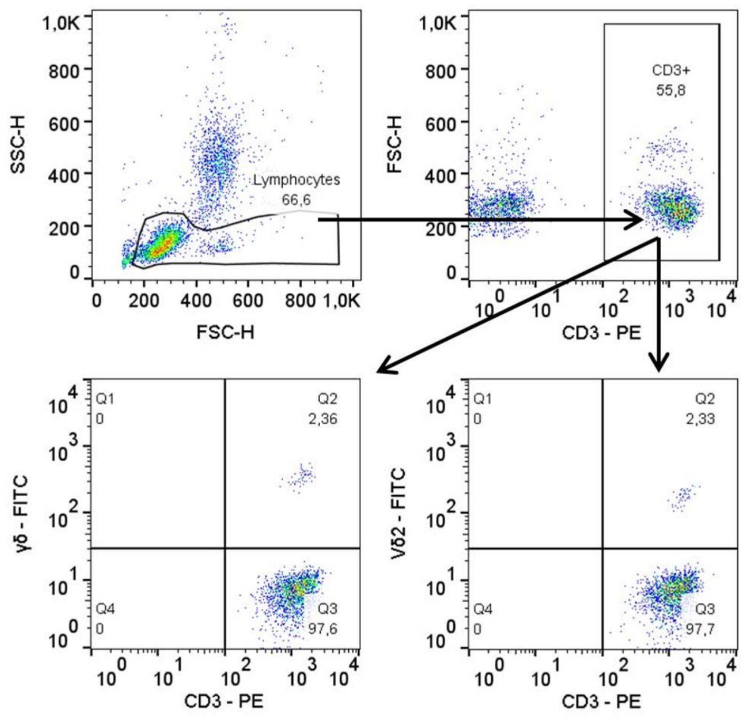

A gate was set on lymphocytes based on forward scatter (FSC) and side scatter (SSC) (upper left). Next, $CD3^+$ T cells were identified (upper right), and $CD3^+$ T cells were then gated according to CD3 and TCRγδ expression (lower left) and CD3 and Vδ2 expression (lower right).

Supplemental Figure S2:

Mean values of total γδ T cells and Vδ2 T cells in retrospective analysis

(a)

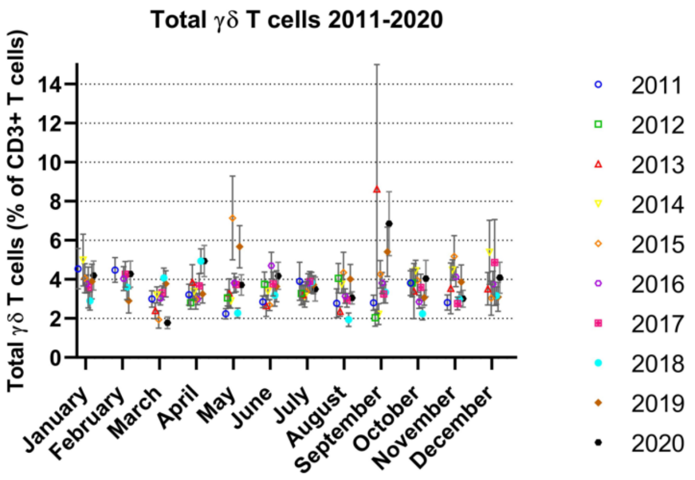

(b)

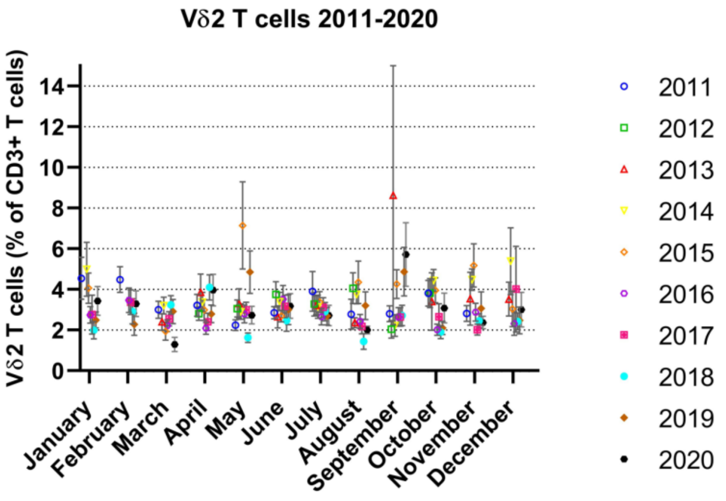

Mean values ± SEM of the proportion of total γδ T cells (a) and Vδ2 T cells (b) within CD3 T cells in all donors according to the month of analysis. The total number of analyses in each year was: 2011: 210, 2012: 69, 2013: 100, 2014: 262, 2015: 218, 2016: 474, 2017: 418, 2018: 334, 2019: 270, 2020: 270

Supplemental Figure S3:

Gating strategy for prospective analysis

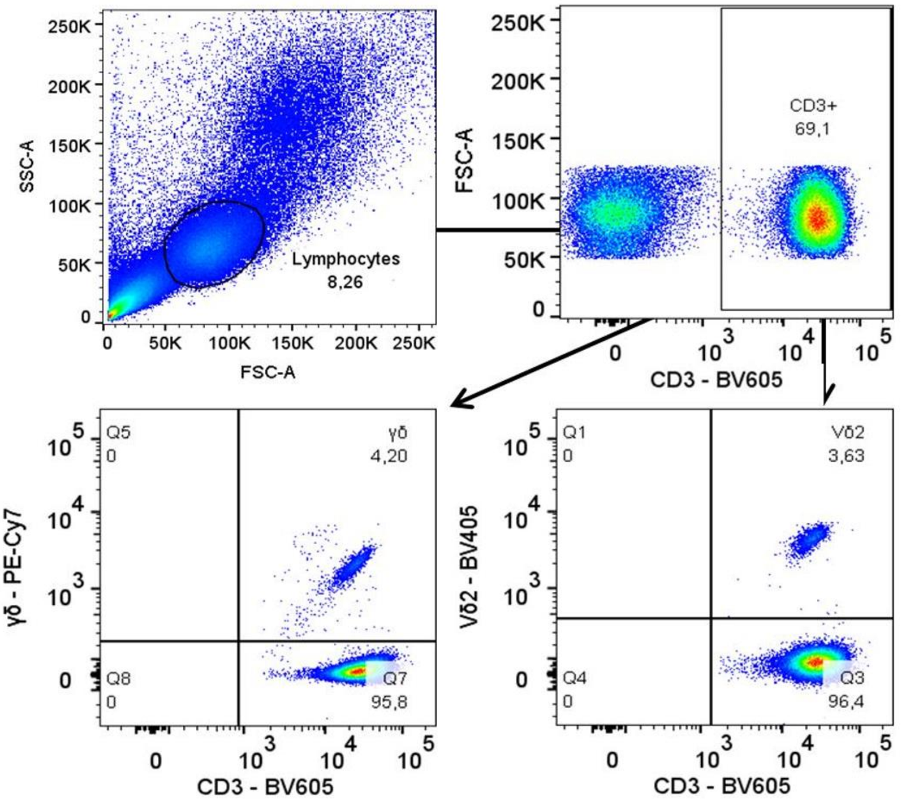

A gate was set on lymphocytes in whole blood based on forward scatter (FSC) and side scatter (SSC) (upper left). Next, $CD3^+$ T cells were identified (upper right), and $CD3^+$ T cells were then gated according to CD3 and TCRγδ expression (lower left) and CD3 and Vδ2 expression (lower right).

Supplemental Figure S4:

Correlation of Vδ2 T cells with serum 25(OH)D_3 levels

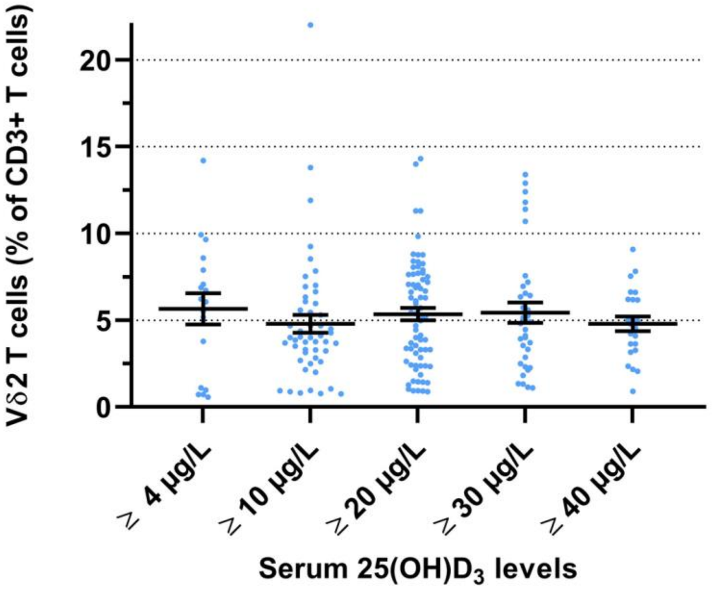

The proportion of Vδ2 T cells among $CD3^+$ T cells of each individual donor in the prospective study was plotted against the serum level of 25(OH)D_3 measured at the same day. The levels of 25(OH)D_3 were grouped as indicated on the Y axis.

Supplemental Figure S5:

Seasonal analysis of non-Vδ2 γδ T cells and correlation with Vitamin D_3 in the prospective analysis

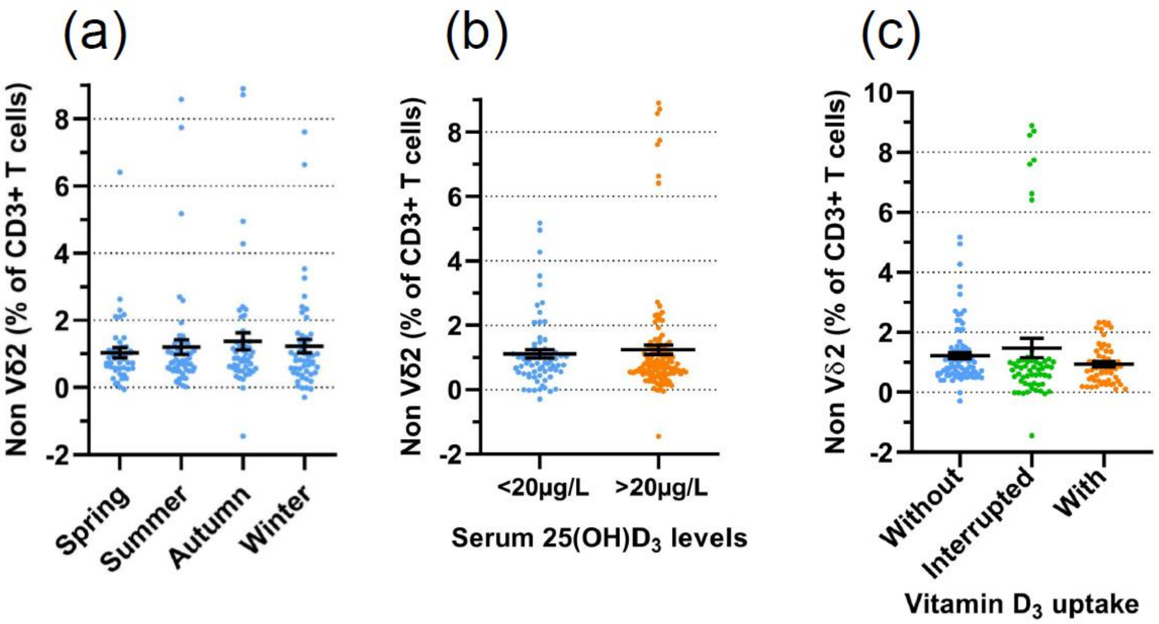

Non-Vδ2 γδ T cells (mainly comprising Vδ1 γδ T cells) were identified as $CD3^{+}TCR\gamma\delta^{+}V\delta2^{-}$. The proportion of non-Vδ2 γδ T cells among CD3 T cells was analyzed according to the season in all donors of the prospective study (a). The proportion of non-Vδ2 γδ T cells was also analyzed in correlation to serum levels of 25(OH)D_3 (b) and to oral Vitamin D_3 supplementation (c). The definition the three groups in (c) is the same as in Figures 2 and 3 of the main manuscript.

Supplemental Figure S6:

Seasonal variation of γδ T cells:

correlation with serum 25(OH)D_3 levels

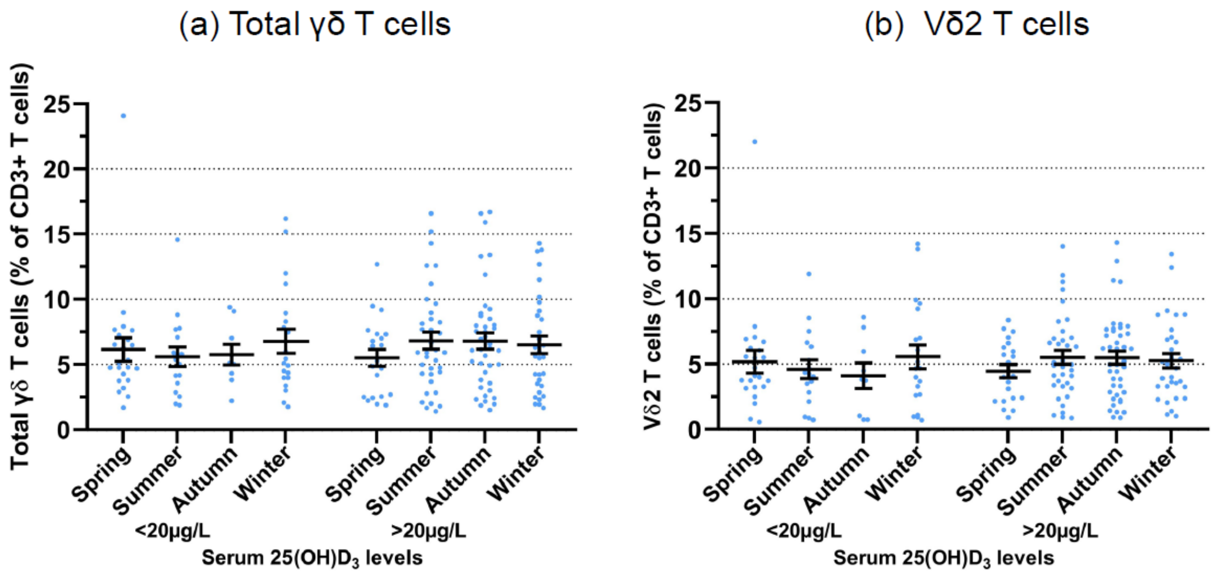

Proportions of total γδ (a) and Vδ2 T cells (b) among $CD3^+$ T cells were measured in blood samples from all donors in the prospective study. Mean values + SEM and individual data points are displayed according to the season and according to the serum level of 25(OH)D_3: low (left panels: < 20 µg/L), normal (right panels: > 20 µg/L).

Supplemental Figure S7:

Growth inhibition of Zoledronate-activated PBMC by 1α,25$(OH)_2D_3$

(a) Macroscopic inspection

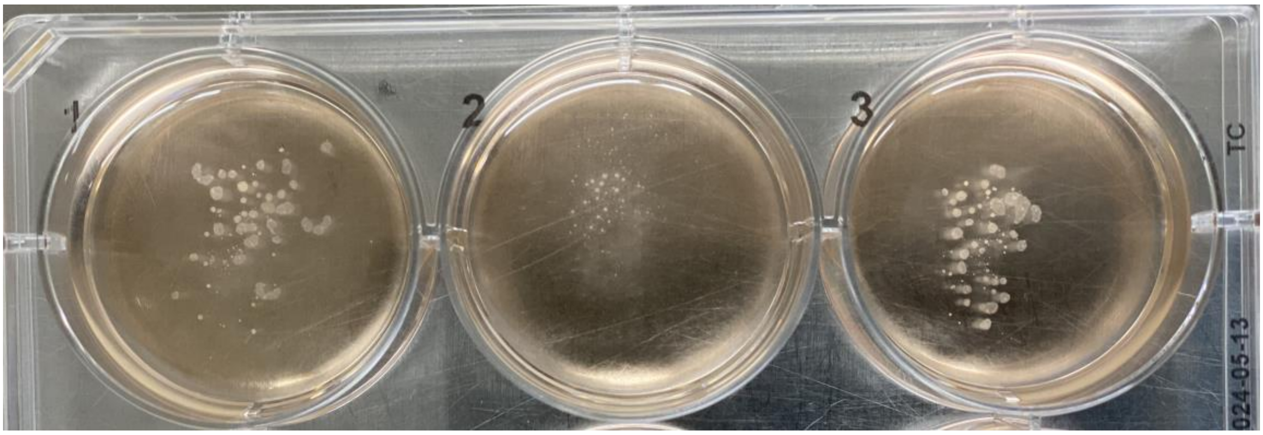

ZOL ZOL + 50nM 1α,25$(OH)_2D_3$ ZOL + DMSO 1:1000

(b) Microscopic pictures (x100)

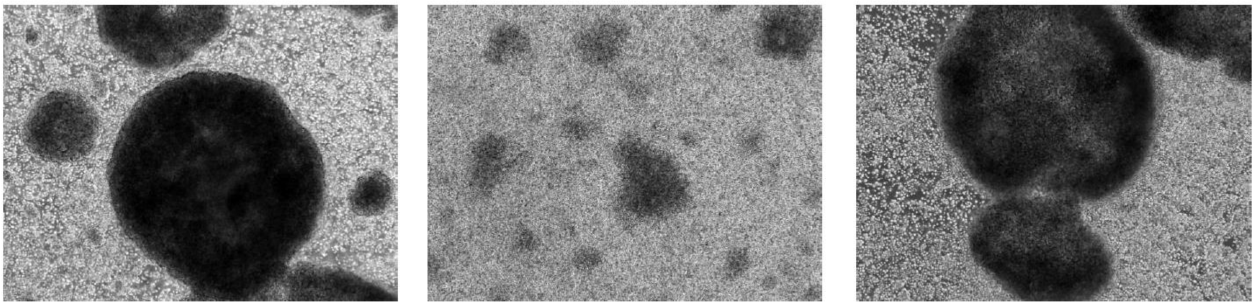

ZOL ZOL + 50nM 1α,25$(OH)_2D_3$ ZOL + DMSO 1:1000

PBMC were cultured at $1x10^6$ cells/mL in 6-well plates (5 mL per well) in the presence of 2.5 µM zoledronate (ZOL) plus 50 IU/mL IL-2 and 50 nM 1α,25$(OH)_2D_3$ or corresponding solvent control DMSO (1:1000). Pictures were taken after 6 d. (a) Macroscopic inspection; (b) microscopic pictures at 100x magnification

Supplemental Figure S8:

Influence of 1α,25(OH)$_2$D$_3$ on cell death of Vγ9 T cells

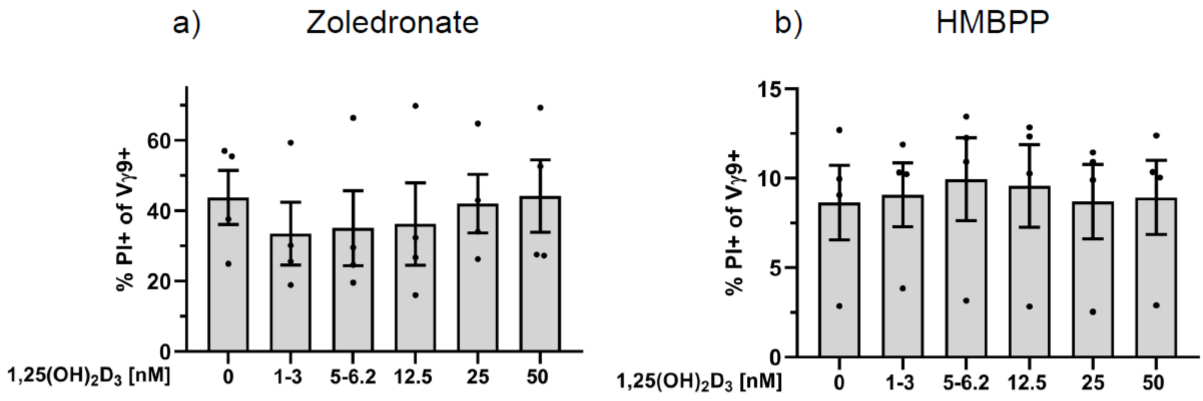

PBMC were stimulated with 2.5 µM ZOL (a) or 10 nM HMBPP (b) in the absence or presence of titrated concentrations of 1α,25(OH)$_2$D$_3$ as indicated. After 8 d, the number of viable Vγ9 T cells was determined by SCDA (see Figure 5). In parallel, the proportion of dead (PI+) Vγ9 T cells was analyzed. Mean values + SEM of four independent experiments are shown.
